# Increased expression of inflammasome signaling genes and proteins in selective brain regions in the intermediate stage of Alzheimer's disease

**DOI:** 10.1111/bpa.70086

**Published:** 2026-02-22

**Authors:** Juan Pablo de Rivero Vaccari, David A. Davis, Andrew P. Sawaya, Susanna P. Garamszegi, Xiaoyan Sun, Ayled Barreda, Helen M. Bramlett, Sakir Humayun Gultekin, W. Dalton Dietrich, Robert W. Keane, Regina T. Vontell

**Affiliations:** ^1^ Department of Neurological Surgery and The Miami Project to Cure Paralysis University of Miami Miller School of Medicine Miami Florida USA; ^2^ Department of Cellular Physiology and Molecular Biophysics University of Miami Miller School of Medicine Miami Florida USA; ^3^ Department of Neurology University of Miami Brain Endowment Bank, University of Miami Miller School of Medicine Miami Florida USA; ^4^ Department of Neurology University of Miami Miller School of Medicine Miami Florida USA; ^5^ Dr. Phillip Frost Department of Dermatology & Cutaneous Surgery University of Miami Miller School of Medicine Miami Florida USA; ^6^ Bruce W. Carter Department of Veterans Affairs Medical Center Miami Florida USA; ^7^ Department of Pathology University of Miami Miller School of Medicine Miami Florida USA; ^8^ Center for Cognitive Neuroscience and Aging, University of Miami Miller School of Medicine Miami Florida USA

**Keywords:** absent in melanoma‐like receptor (ALR), Alzheimer's disease, apoptosis‐associated speck like protein containing a caspase recruitment domain (ASC), gasdermin D (GSDMD), inflammasome, nucleotide‐binding and oligomerization domain (NOD)‐like receptor (NLRP)

## Abstract

Neuritic plaques (NP) are a key component of Alzheimer disease (AD) pathology composed of dystrophic neurites that form neurofibrillary tangles (NFTs) and amyloid‐*β* (A*β*) proteins. NP accumulation is one fundamental feature seen in the intermediate stage of AD neuropathological pathology change. NPs are sites for cellular degeneration and can induce the upregulation of inflammatory signals including the inflammasome complex. We analyzed 758 genes using multiplex genomics in samples from the hippocampal, temporal and frontal brain regions that had intermediate AD neuropathological changes and aged‐matched controls. In addition, we analyzed NP formation using *phosphorylated tau at threonine 217* (pTau217) and A*β* antibodies along with inflammasome sensors using NOD‐like receptor protein (NLRP) antibodies, *apoptosis‐associated speck like protein containing a caspase recruitment domain* (ASC) and *absent in melanoma‐like receptor 2* (AIM2). Finally, we investigated if cell death is occurring cells by pyroptosis using Gasdermin D (GSDMD) antibody to detect pore formation on the membrane. Analyses show that inflammasome signaling genes and proteins are significantly increased in the hippocampus rather than the temporal and frontal lobe at the intermediate stage of AD. NPs are more prevalent in the hippocampus and temporal lobe in cases with intermediate AD pathology. ASC (1C100) immunostaining is colocalized with NLRP1, NLRP3 and AIM2. GSDMD immunostaining detected pericellular pores forming around the membrane of NFTs and in NPs. Messenger RNA and protein analyses demonstrate that inflammasome signaling molecules are elevated in AD suggesting that neuronal death occurs by the induction of pyroptosis.

## Graphical abstract

Neuritic plaques increase in the intermediate stage of Alzheimer's neuropathological change. The intermediate stage of Alzheimer's disease was investigated by transcriptomics and immunohistochemistry. This revealed that inflammasome sensors NLRP1, NLRP3, and AIM2 oligomerize with ASC speck to form the inflammasome complex and initiate the downstream signaling of GSDMD‐mediated pyroptosis.

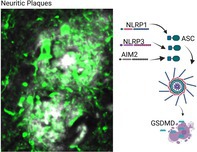

## 1 INTRODUCTION

Alzheimer's disease (AD) is a clinical syndrome characterized by progressive cognitive decline. Histologically, AD has neuropathological changes that include extracellular amyloid‐*β* (A*β*) plaques and intracellular hyperphosphorylated tau neurofibrillary tangles (NFTs).^1^ Another pathologic feature, neuritic plaques (NP), contains hyperphosphorylated tau that presents in the form of dense dystrophic neurites which are surrounded by a core of A*β* aggregates.^2^ NPs cause dysregulation of neuronal networks,^3^, ^4^ and induce chronic neuroinflammatory signals by releasing numerous cytokines^5^ and activating microglia^6^ all of which can exacerbate neuropathological changes.

A key contributor to the inflammatory response in AD is the formation of inflammasomes which play a critical role in the early stages of cognitive changes^7^, ^8^, ^9^ and also the later stages in which A*β* activates inflammasome proteins.^8^, ^10^ The inflammasome formation is triggered via the activation of sensors such as the nucleotide‐binding and oligomerization domain (NOD)‐like receptors (NLRs) NLRP1 or NLRP3^11^ or absent in melanoma‐like receptor 2 (ALR) such as AIM2 followed by oligomerization with ASC and caspase‐1 to cleave caspase‐1, followed by the processing of IL‐1 cytokines.^12^ Both NLRs and AIM2 are an intricate part of pyroptosis signaling^13^ and the inflammasome in neurons.^14^ A non‐canonical inflammasome pathway is via caspase‐8 activation, which normally functions as an extrinsic apoptotic signaling and prevents necroptotic cell death, but it also can induce pyroptotic cell death.^15^, ^16^ NLRs, AIM2 and caspases oligomerizes with apoptosis‐associated speck like protein containing a caspase recruitment domain (ASC) to cleave caspase‐1 and induce processing of interleukins (IL), namely IL‐1*β*, IL‐18 and/or pyroptotic cell death.^17^, ^18^ Pyroptosis is induced by the protein signaling of Gasdermin D (GSDMD), when it is cleaved by caspase‐1 at the N terminal fragment. GSDMD programmed cell death results in 1–2 nm pores to form in the plasma membrane that causes the cell to lyse and release proinflammatory contents.^18^

The formation of A*β* plaques and NFTs progress alongside a host of neuroinflammatory cascades in the brain, and activate a diverse population of cell types, most notably microglial and astroglia,^19^ Microglia possess receptors that respond to A*β* plaques, nanoparticles, NPs, and apolipoprotein E (APOE). Presenilin 1 is thought to regulate the cellular levels of triggering receptor expressed on myeloid cells 2 (TREM2) on microglia; upon activation, TREM2 induces the phagocytic uptake of extracellular A*β* components.^12^ Deficiency of TREM2 impairs the phagocytic capacity of damaged‐associated microglia, which in turn aggravates the pathogenic response and increases inflammasome formation in neurodegenerative disorders, such as AD and Parkinson's related dementias.^12^, ^20^

AD neuropathological changes are assessed in three stages, low, intermediate, and high AD. These changes integrate the scoring the quantity and the location of A*β* plaques (Thal staging, “A” score), NPs (CEREAD staging, “C” score), and NFTs (Braak Score, “B” score), which can be correlated with the patient clinical history.^4^, ^21^ Low AD neuropathological stage, which is characterized by low NFT populations with and without A*β* plaque formation and a few NPs identified in the hippocampus, amygdala and frontotemporal neocortical regions,^22^ In these cases, the hallmark of AD histological changes of NFTs are present mainly in the amygdala and the hippocampus (e.g., Braak I‐II), but the low densities of NFTs are considered to be age‐related changes or preclinical AD.^23^ In contrast, in intermediate neuropathological AD staging, a severely affected amygdala and hippocampus is present with a heavy burden of A*β*, NFTs, NPs and reactive microglia. Other neocortical regions show AD neuropathological changes and display a varying amounts hyperphosphorylated tau, A*β* and NPs^24^, ^25^ (e.g., Braak III‐V) along with increased microglia and inflammasome proteins.^8^ In addition, other vulnerable brain regions such as the medial temporal gyrus shows moderate amounts of NFTs, while the superior frontal gyrus has sparse densities of NFTs.^26^ Furthermore, PET scan studies suggest that as tau deposition spreads to the medial temporal region, there is a spread of neuroinflammatory modulators.^26^, ^27^ Clinically, donors assed at the intermediate AD neuropathological stage presented a history of mild cognitive impairments (MCI) to moderate impairment or even severe cognitive changes.^28^ Moreover, high AD neuropathological change is characterized by NFTs that spread to the parastriate cortex and occipital lobe (Braak V‐VI). In conjunction with the NFTs are higher densities of Aβ plaque formations, severe amounts of NPs are identified in multiple brain regions, along with active microglia and reactive astrogliosis.^29^ It is thought that patients with an early onset of MCI (younger than age 65) are at a greater risk of quickly progressing to the intermediate and high AD stages.^30^, ^31^, ^32^, ^33^ Therefore, identifying how the propagation of inflammation occurs from one brain region to another is key to developing therapies to prevent or slow down progressive dementia.^27^

The number of individuals with AD neuropathological changes is projected to increase, especially in individuals over age 85 and as rates of longevity increase.^30^ Women are at a higher risk of developing AD related dementia due to increased life expectancy and biological factors.^34^ Although men are at risk for vascular comorbidities that correlate with cognitive decline and AD, in women with cardiovascular disease, the incidence of AD related dementia is higher.^34^ Because of increasing prevalence of AD related dementias seen in the ageing population, it is imperative that proposed biomarkers and therapeutic targets are investiaged.^23^ Phosphorylated tau at threonine 217 (pTau217) has recently been shown to have consistent and high performance in detecting AD pathology in patients with MCI and differentiates between other neurodegenerative disorders.^35^, ^36^, ^37^ Higher plasma pTau217 concentration correlates with by increasing amounts of Aβ plaques, tau tangles,^37^ and NPs identified in neuropathological assessment,^2^, ^4^ however, the relationship between pTau 217 accumulation and inflammatory responses remains unknown. Early biomarkers markers, such as pTau 217, are essential for detecting MCIs and monitoring dementia progression in AD patients. Beyond its diagnostic utility, pTau217 is a vital tool for evaluating the efficacy of emerging therapeutics designed to slow symptom development. Recent evidence suggests that pTau217 may directly trigger NLRP3 inflammasome activation, which in turn drives further tau phosphorylation and neuroinflammation. Currently, NLRP3 inhibitors are being tested as a target to treat inflammatory diseases in humans and mouse AD models demonstrate that NLRP3 induce inflammasome pathway exacerbates amyloid pathology and tauopathies.^38^, ^39^ Recently, we identified ASC formation in postmortem brain samples of donors with Parkinson's disease, and we showed that the treatment of LRRK2 cells with ASC specks induced inflammasome activation and cytotoxicity.^40^ Consequently, investigating the association between pTau217 and inflammasome assembly in postmortem samples at intermediate AD stages is critical. This approach helps validate the inflammasome as a viable therapeutic target and provides a measurable baseline for identifying the efficacy of anti‐inflammatory interventions in AD.^41^, ^42^ Thus, comprehending how pTau217 associates with the inflammasomes in postmortem samples at the intermediate stage of AD has the benefit of identifying the therapeutic efficacy of this multiprotein complex of the innate immune response as a potential target for AD.

This study investigates the tissue expression pattern of inflammatory mechanisms involved in the neurodegenerative processes between low and intermediate AD in three different brain regions. We hypothesized that the inflammatory gene expression should differ in highly affected brain regions compared to regions known to have minimal pathological change at the intermediate AD stage. In addition, we aimed to investigate the relationship between pTau217 and inflammasome formation in neurons and NPs that form at the intermediate AD pathological stage.

## 2 METHODS

### 2.1 Postmortem human brains

Informed consent was acquired for permitting postmortem research examination according to the University of Miami, following the Institutional Regulatory Board (IRB) guidelines. Research study ethics was obtained from the Human Subjects Research Office at the University of Miami, Miami, Florida (IRB ethics number 19920348 (CR00012340) Brain Endowment Bank).

### 2.2 Tissue preparation

Case demographic details including age, cognitive status, postmortem interval tissue, and neuropathological analyses have already been described in Reference [8] and are summarized in Supplemental Data 1. The regions of interest (ROI) included fresh frozen samples from the left hemisphere and formalin fixed samples from the right hemisphere taken from the frontal lobe (BA9), the temporal lobe (BA21), and the hippocampus. Twelve of the brains showed age‐related AD neuropathological changes (low AD; Braak none‐stage II) on gross and microscopic examination and were used as non‐neuropathological controls (control cases). The remaining 12 brains showed intermediate AD neuropathological changes (using A*β* and pTau (AT‐8) immunohistochemistry) identified to be at Braak stages III–V after review of pathological examination using the guidelines described in Reference [21].

### 2.3 RNA extraction

Total RNA was extracted from approximately 100 mg of fresh frozen tissue using RNeasy Lipid Tissue Mini Kit (#74804; Qiagen Inc., Valencia, CA, USA) with on‐column DNase I treatment to eliminate genomic DNA contamination. RNA concentration was measured with a NanoDrop™ 2000 (Wilmington, DE, USA), and the quality was determined based on 260 and 280 nm absorbance wavelengths. The ratio of ~2.0 is considered pure for RNA.

### 2.4 NanoString

Experiments were conducted to test the gene expression measured on the NanoString nCounter (NanoString, Seattle, WA, USA) in the three different brain regions (BA9, BA21, and hippocampus). NanoString probes were designed to match the classifier genes and 13 housekeeper genes from the Alzheimer's Panel and a few selected genes from the Glial Profiling Panels. The NanoString nCounter® was conducted using NanoString's standard life sciences custom CodeSets, consumables, and assay procedures. The NanoString gene expression measurements were assessed for all target genes and housekeeping genes using 24 samples from archived fresh frozen tissue samples from each ROI. RNA input for the samples was 100 ng (per NanoString reaction) for each probe. Gene analysis was conducted using nSolver™ 4.0 Software (NanoString) with an adjusted *p‐*value of 0.05 or less considered significant. Volcano plots of differential gene expression were made using SRPLOT software (Shanghai Jiao Tong University, Xiangya School of Medicine, Shanghai, China).

### 2.5 Pathway analysis

Pathway analysis was conducted on differentially expressed genes using Ingenuity Pathway Analysis (IPA; Qiagen, USA), which yielded enrichment *p*‐values and predicted activation (*z*‐scores) for canonical pathways, biological processes, and upstream regulators. IPA software used Fishers exact test to detect significantly enriched pathways and biological processes with *p*‐values 0.05 were considered significant.

### 2.6 Immunofluorescence labeling

To identify the cellular location of the NP protein in our tissue immunofluorescence (IF) labeling was performed using IF protocols described in Reference [8]. In brief, to identify the cellular location of the NPs proteins in our tissue IF triple‐labeling was performed on the intermediate AD and on the controls with low AD pathological changes. The primary antibodies were compatible and mixed as a cocktail of rabbit anti‐phospho‐Tau (Thr217;2.0 μg/mL; Invitrogen; catalog# 44744; Carlsbad, CA, USA), human anti‐ASC (IC100, 2 μg/mL; ZyVersa Therapeutics, LLC. Weston, FL, USA), and mouse anti‐beta amyloid 1–16 (1.0 μg/m; BioLegend;SIG‐39155; San Diego, CA). Mouse anti‐NLRP3 (3.0 μg/mL MilliporeSigma; HPA01287; Burlington, MA, USA), human anti‐ASC (IC100, 2 μg/mL) and Rabbit anti‐NLRP1 (1.0 μg/mL; Enzo Life Sciences; Farmingdale, NY, USA). Mouse anti‐NLRP3 (3.0 μg/mL MilliporeSigma; HPA01287; Burlington, MA, USA), Rabbit anti‐Glial Fibrillary Acidic Protein (GFAP) (1 μg/mL; PA‐10019; MilliporeSigma) and chicken anti‐Ionized calcium‐binding adaptor molecule 1 (Iba1) (0.5 μg/mL; PA5‐14372; MilliporeSigma). Mouse anti‐HuC/HuD (16A11; 1.5 μg/mL A‐21271; MilliporeSigma), mouse anti‐Neurofilament (NF) (NAP4; 0.5 μg/mL MA‐10041; MilliporeSigma), chicken anti‐Iba‐1 (0.5 μg/mL; PA5‐14372; MilliporeSigma) and rabbit anti‐NLRP1 (1.0 μg/mL; Enzo Life Sciences). Rabbit anti‐AIM2 (1.0 μg/mL Invitrogen; catalog# BS‐5986R), human anti‐ASC (IC100, 2 μg/mL) and mouse anti‐NLRP1 mouse anti‐NLRP1 (1.0 μg/mL; Enzo Life Sciences, USA). Mouse anti‐beta amyloid 1–16 (1.0 μg/m; BioLegend;SIG‐39155), mouse anti‐phospho‐Tau (Ser202, Thr205) (AT8; 0.2 μg/mL 4744‐RBM4‐P1; MilliporeSigma), rabbit anti‐GSDMD (1D11; 3.0 μg/mL ZRB1274; MilliporeSigma) and chicken anti‐Iba‐1 (0.5 μg/mL; PA5‐14372; MilliporeSigma).

Sections were pretreated with 5 min in formic acid and 20 min in 10 mM preheated citric acid (Millipore) and cooled, then blocked in 5% goat serum for 20 min before the primary antibodies were applied and incubated overnight at 4°C. Following primary antibody incubation, sections were rinsed three times in PBS for 3 min each time before these secondary antibodies were added. The samples were finally soaked for 1.5 h in PBS containing the following secondary antibody cocktail: goat anti‐rabbit IgG conjugated to Alexa Fluor 488 (4 μg/mL; Invitrogen), goat anti‐human IgG conjugated to Alexa Fluor 546 (4 μg/mL; Invitrogen), and goat anti‐mouse conjugated to Alexa Fluor 647 (4 μg/mL; Invitrogen). Finally, the sections were placed under a coverslip using ProLong Gold antifade reagent (Invitrogen) and then kept in the dark at 4°C until analysis.

### 2.7 Neurofibrillary tangles, neuritic plaque and GSDMD assessment

Scoring analysis of A*β* plaques, NPs and NFTs (seen with using p‐Tau 217) in the ROI were determined by the cellular architecture previously as described in References [8, 43]. Unbiased cell counts of A*β* clusters, NPs (formations of A*β* plaques with pTau217 dystrophic neuritic cores), and pTau217 positive neurons were counted in an average area of 1.2 mm^2^ and counts were normalized to 1 mm^2^ area. Scanned images were obtained using an Olympus OlyVIA virtual tissue scan. Contours from each were taken from the CA1 region of the hippocampus, BA9 of the frontal lobe and BA21 of the temporal lobe, which encompassed an average area of 1–2 mm^2^ per region cropped by using the Image‐Pro Premier program (Media Cybernetics, USA). Contours from GSDMD images were taken from the CA2 region of the hippocampus. Mean density luminosity was conducted on the GSDMD positive neuronal cell bodies in an average area of 1.0 mm^2^ cropped by using the Image‐Pro Premier program (Media Cybernetics).

Cellular densities of tau positive‐stained neurons, A*β* clusters and NP in all contours were quantified by investigators who were blinded to case data. Tissue scans were reviewed (by RV) to ensure that counts had met the criteria to avoid duplication of counts (e.g., an area containing a tau positive neuron or A*β* cluster [>10 μm^2^] or NP (positive A*β* core connected positive pTau217 processes) [>20 μm^2^]). In a pilot study, we confirmed that the counting profile described previously counted the correct number of labeled cells and nuclei (using an Image J cell counter).

Estimation of number density was performed by applying the following formula^44^:

$$N=\frac{\Sigma Q^{-}}{V}$$

where *N* is the total number of cells or clusters per volume of brain region; Σ*Q* ^−^ is the number of counted cells; and *V* is the volume of regions of interest per sampling frame.

### 2.8 Immunoblotting

Immunoblot analysis of post‐mortem hippocampal lysates (*n* = 12) (Lysis buffer: Protease Inhibitor Cocktail (Sigma Aldrich #P8340), 20 mM Tris HCl, 150 mM NaCl, 1 mM EDTA, 1 mM EGTA, Triton X‐100, 2.5 mM Na_4_P_2_O_7_, 1 mM Na_3_VO_4_, 1 mM *β*‐glycerophosphate) from decedents between the ages of 67–94 corresponding to Braak stages I (controls) to IV (intermediate AD) were carried out as described in Reference [45] using a primary rabbit antibody against AIM2 (Novus Biologicals, Cat#NBP2‐15313; Centennial, CO., USA) at 1:1000 dilution followed by HRP‐linked IgG secondary antibody against rabbit (1:1000, Cell Signaling, Cat#7074S; Davers, MA., USA).

### 2.9 Data analysis

A non‐parametric test was performed to compare the variables between two groups (Intermediate AD and Controls) in all experiments. Prior to the testing for statistical test normality distribution of data and variance homogeneity were checked by making a Q–Q plot of the data and using the Shapiro–Wilk test. Comparison between the two groups was conducted using a Mann–Whitney test for data, as the Shapiro–Wilk tests showed that the data were not normally distributed in all the experiments across the 3 brain ROIs included in this study. Data are presented as mean ± SD; significance was assumed at *p* <0.05. Analysis was used to test the difference between the number of A*β* diffuse plaques, NPs, NFT numbers (pTau 217), and ASC accumulation as described for each of the ROI's. All statistical analyses and generation of graphs were performed using GraphPad Prism 9.0 (GraphPad Software, San Diego, CA, USA). Data were presented as mean ± standard deviation (SD); significance was assumed at *p* <0.05.

## 3 RESULTS

Experiments in this study were designed to evaluate the mRNA expression using the NanoString nCounter® AD panel and the Glial profiling panel. We were able to evaluate over 758 genes in each ROI (the hippocampus CA1 region, as well as the BA21 and the BA9 brain regions) in postmortem brain tissue with and without intermediate AD pathology. Of these genes, 440 were related to inflammatory processes. We then investigated apolipoprotein E, (*APOE*) AD specific genes, post‐synaptic genes, neurogranin (*NRGN*). and inflammatory genes in human postmortem brain regions from the hippocampus, and the temporal and frontal lobes in control and cases with intermediate AD neuropathology. Additionally, we sought to find sex differences in gene expression in the hippocampus, BA21 and BA9 regions. Finally, we investigated the cell type distribution and expression of the inflammasome signaling proteins in hippocampal brain regions in cases of intermediate AD neuropathology using multiple immunofluorescent labeling approaches. (Neuropathological scores and demographics are reviewed in Supplementary Table 1.)

### 3.1 Gene patterns differ in different brain regions

**FIGURE 1:**
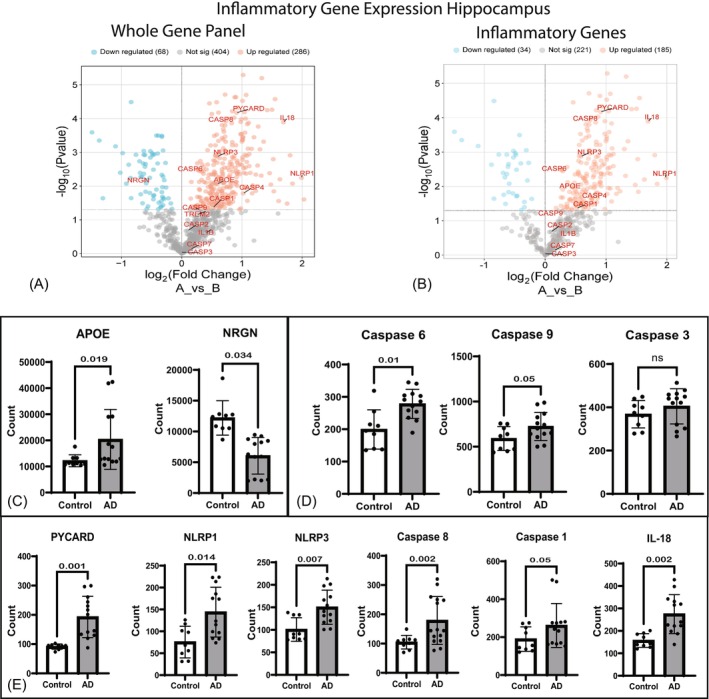
Multiplex gene expression from hippocampal RNA extracted from the hippocampus CA1 region of control and intermediate AD donors. (A) Whole gene RNA counts. (B) Inflammation‐specific genes. (C) Increase in *APOE* RNA and decrease in neurogranin (*NRGN*) gene expression. (D) Upstream mediators of inflammatory initiators (*caspase‐6* and *caspase‐9*) significantly differed between controls and intermediate AD but not the downstream initiators of apoptosis, *caspase‐3*. (E) Inflammasome complex gene expression showed significant upregulation of *caspase‐1*, *PYCARD*, *NLRP1*, *NLRP3* and *caspase‐8* genes and an increase in cytokine *IL‐18* indicating that pyroptosis signaling is involved in cell death in intermediate AD cases. Cornu Ammonis Field 1 (CA1); ribonucleic acid (RNA); Alzheimer's disease (AD); Apolipoprotein E (APOE); Apoptosis‐associated Speck‐like protein containing a CARD (PYCARD); NOD‐like receptor protein (NLRP); interleukin (IL); ns = not significant; adjusted *p*‐values are shown.

At the intermediate stage of AD neuropathological change, the hippocampus has a significant increase in tauopathies, A*β* clusters, and NPs which relate to an increase in the inflammatory response. There was a significant upregulation of 286 genes and a downregulation of 68 genes in the intermediate AD group compared to the controls (Figure 1A) in the hippocampus. Moreover, 185 genes related to inflammatory processes out of 440 were upregulated and 34 were shown to be downregulated (Figure 1B). *APOE* was upregulated in the intermediate AD cases, whereas *NRGN* was downregulated (Figure 1C). *Caspases‐6* and *‐9* were upregulated in the hippocampus in the intermediate AD cases, but there was no change in *caspase‐3* mRNA level (Figure 1D). The data showed no significant change in *TREM2* expression between intermediate AD and controls. The inflammasome pathway gene expression in the hippocampus significantly increased in intermediate AD cases. Accordingly, *caspase‐1, PYCARD*, *NLRP1*, *NLRP3*, *caspase‐8*, as well as *IL‐18* (Figure 1E) mRNA levels increased in the intermediate AD group indicating that in the hippocampus there is an increase in genes that favor inflammasome signaling (See Supplementary Data 1 Table 2).

The data were separated by sex to investigate how the gene expression in females and males with intermediate AD cases compares with controls of the same sex in the hippocampal region. In females, we found that 40 genes were downregulated, 209 were upregulated and 509 did not significantly change in females with intermediate AD compared to control females. Of those, *APOE* was upregulated in the female donors with intermediate AD cases, whereas *NRGN* was downregulated. *Caspases‐6*, *‐9* and *caspase‐3* mRNA levels did not change in the female intermediate AD group compared to the female controls. However, the mRNA levels of *TREM2* and inflammasome genes *caspase‐1*, *PYCARD*, *NLRP1*, *NLRP3*, *caspase‐8*, as well as *IL‐18* were upregulated in the intermediate AD group (Supplementary Figure 2A,C). In males, we found that 52 genes were downregulated, 83 genes were upregulated and 623 genes did not significantly change in males with intermediate AD compared to male controls. Of interest, the males with intermediate AD had no change in *NRGN* or *APOE* mRNA levels. *Caspase‐6* was upregulated in the hippocampus in the males with intermediate AD, but there was no change in *caspases‐3*, *‐4*, *‐8*, *and ‐9*. *TREM2* mRNA levels were not significantly changed. The mRNA reading of inflammasome genes *caspase‐1*, *PYCARD*, *NLRP1*, *NLRP3*, as well as *IL‐18* did show an increase in expression in the male intermediate AD (Supplementary Figure 2B,C).

**FIGURE 2:**
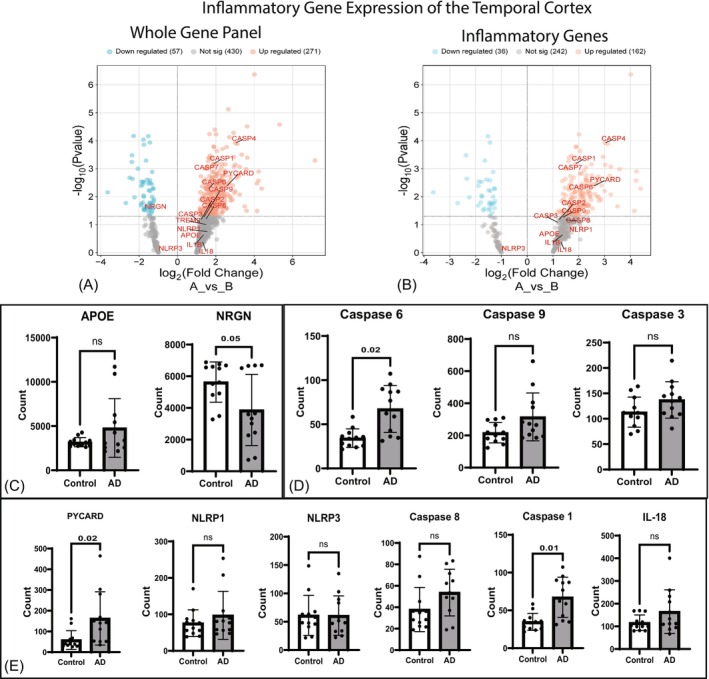
Multiplex gene expression from temporal lobe (BA21) RNA extracted from controls and intermediate AD donors. (A) Whole gene panel. (B) Inflammation‐specific genes. (C) No significant changes in *APOE* but neurogranin (*NRGN*) RNA counts were shown to decrease between intermediate AD and controls. (D) Common upstream mediators of inflammatory initiators (*caspases‐6*) significantly increased in intermediate AD cases, but without downstream mediation of apoptosis (*caspase‐3*). (E) Inflammasome complex gene expression showed significant upregulation of *caspase‐1* and *PYCARD* but not a significant difference in *NLRP1*, *NLRP3*, and *IL‐18* genes. Ribonucleic acid (RNA); Alzheimer's disease (AD); Apolipoprotein E (APOE); Apoptosis‐associated Speck‐like protein containing a CARD (PYCARD); NOD‐like receptor protein (NLRP); interleukin (IL); ns = not significant; adjusted *p*‐values are shown.

In neuropathological staging of AD, tau pathology spreads to the medial temporal lobe by Braak stage IV, which includes BA 21^8^, ^25^ and clinical reviews that the donor had a decline in cognitive scores and executive function.^46^ In BA21 we evaluated 758 mRNA levels identified in the Alzheimer's and Glial NanoString Panels to investigate whether this brain region presented similar RNA changes. In BA21 there were 271 genes upregulated, 57 genes downregulated, and 430 genes did not change between intermediate AD and controls (Figure 2A). Comparing the mRNA counts for the 440 genes involved in inflammation, 162 genes significantly increased, and 36 were downregulated in intermediate AD cases (Figure 2B). The gene expression for *APOE* did not significantly change between control and intermediate AD cases, but there was a significant decrease seen in neurogranin expression in the intermediate AD (Figure 2C). Inflammatory initiators *caspase‐6* showed significant increases, but *caspase‐9*, *‐3* did not significantly differ between control and intermediate AD (Figure 2D). The inflammasome gene expression significantly increased in *PYCARD* and *caspase‐1*, but the gene expression of *NLRP1*, *NLRP3*, *caspase‐8* and *IL‐18* did not significantly change (Figure 2E).

When we separated the gene readings of the temporal lobe by sex to investigate how the gene expression differs in females and males with intermediate AD cases. We found higher rate of change in females. In females, we found that 58 genes were downregulated, 304 were upregulated, and 396 did not significantly change with intermediate AD compared to control females. Of those, *APOE* was upregulated in the female donors with intermediate AD cases, whereas *NRGN* was downregulated. *Caspases‐4 and ‐6* were upregulated, and caspases‐3 and ‐*9* mRNA levels did not change in the female intermediate AD group. The mRNA levels of *TREM2* and inflammasome genes *caspase‐1*, *PYCARD*, and *caspase‐8* were upregulated, whereas *NLRP1*, *NLRP3*, and *IL‐18* did not show a change in expression in the female intermediate AD group (Supplementary Figure 3A,C). Of interest, the males with intermediate AD had no change in *NRGN* or *APOE* mRNA levels. *Caspases‐6*, *‐3*, *‐4*, *‐8*, *and ‐9* did not show a difference in mRNA expression in the temporal lobe in the males with intermediate AD. There was no change in *TREM2* mRNA levels or in the inflammasome genes *PYCARD*, *NLRP1*, *NLRP3*, and *IL‐18*. Of interest, *caspase‐1* did show an increase in mRNA levels in the temporal lobe of the males with intermediate AD group compared to male controls (Supplementary Figure 3B,C).

**FIGURE 3:**
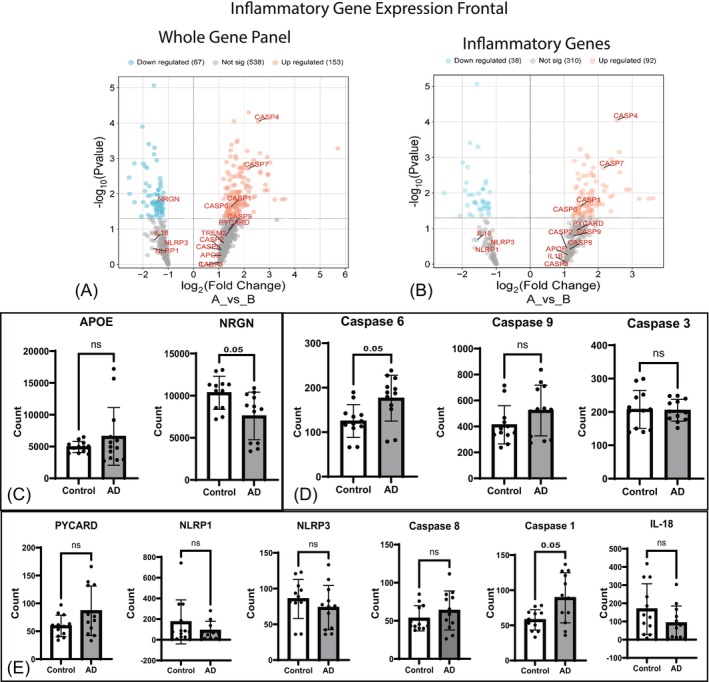
Multiplex gene expression from frontal lobe (BA9) RNA extracted from control and intermediate AD donors. (A) whole gene panel. (B) Inflammatory specific genes. (C) No significant changes in *APOE* RNA were present, however there was a decrease in neurogranin (*NRGN*) RNA. (D) Upstream mediators of initiator (*caspase‐6*) significantly differed between controls and intermediate AD, but not *caspase‐9* or downstream mediator *caspase‐3*. (E) Inflammasome complex gene expression showed significant upregulation of *caspase‐1*, but not a significant change in *PYCARD*, *NLRP1* and *NLRP3* genes or *IL‐18*. Ribonucleic acid (RNA); Alzheimer's disease (AD); Apolipoprotein E (APOE); Apoptosis‐associated Speck‐like protein containing a CARD (PYCARD); NOD‐like receptor protein (NLRP); interleukin (IL); ns = not significant; adjusted *p*‐values are shown.

The temporal cortex and hippocampus sections are characterized by robust AD neuropathological changes during Braak stages III–IV, but these changes are limited in other regions of the frontal and sensory cortices.^25^ Here, the last region that was studied for RNA expression was the BA9 region of the frontal lobe. The AD RNA panel showed 153 genes upregulated, 67 genes downregulated, and 538 genes were not altered when comparing intermediate AD to controls (Figure 3A). Of the 440 inflammatory genes, 92 genes were upregulated and 38 were downregulated (Figure 3B). Of interest, *APOE* gene expression did not significantly change, but the *NRGN* gene was decreased (Figure 3C). In the intermediate cases, caspase‐6 was significantly increased, but there was no change in *caspases‐9 or ‐3* (Figure 3D). The mRNA expression of *caspase‐1* was increased; however, *PYCARD*, *NLRP1*, *NLRP3*, *caspase‐8* and *IL‐18* levels did not change between the intermediate AD and control groups (Figure 3E). Sex differences showed different significant mRNA expression in females and males with intermediate AD cases compared with controls of same sex in frontal lobe region. In females, we found that 19 genes were downregulated, 89 were upregulated and 650 did not significantly change in females with intermediate AD. There were no changes in mRNA expression of *APOE* or *NRGN* in the intermediate AD female group. *Caspases‐6*, and *‐9* mRNA levels did not change, but of interest caspases‐3 and ‐4 were upregulated in the female intermediate AD group compared to the controls. The mRNA levels of *TREM2* and *PYCARD* mRNA expression were upregulated, but *caspase‐8*, *NLRP1*, *NLRP3* and *IL‐18* did not change expression in the females with intermediate AD group (Supplementary Figure 4A,C). In the males with intermediate AD there was no change in mRNA levels of *NRGN, APOE*, *caspases‐6*, *‐8*, *and ‐9* in the frontal lobe. The inflammasome associated genes *TREM2*, *PYCARD*, *NLRP1*, *NLRP3*, and *IL‐18* did not significantly change in the male intermediate AD group. However, the mRNA levels in caspases‐1, *and ‐3* did show an upregulation in frontal lobe of the males with intermediate AD group compared to male controls (Supplementary Figure 4B,C). Together, these data are consistent with the notion that the propagation of inflammasome genes upregulate as NFTs, A*β* clusters and NP increase.

### 3.2 Neuritic plaques

Since the hippocampus showed the most prominent expression of inflammasome genes, we investigated whether increases in mRNA expression of inflammasome components translate to protein production of the inflammasome complex. PTau217 has been recently shown to be an AD specific tau isoform used to detect AD in blood and CSF.^35^

**FIGURE 4:**
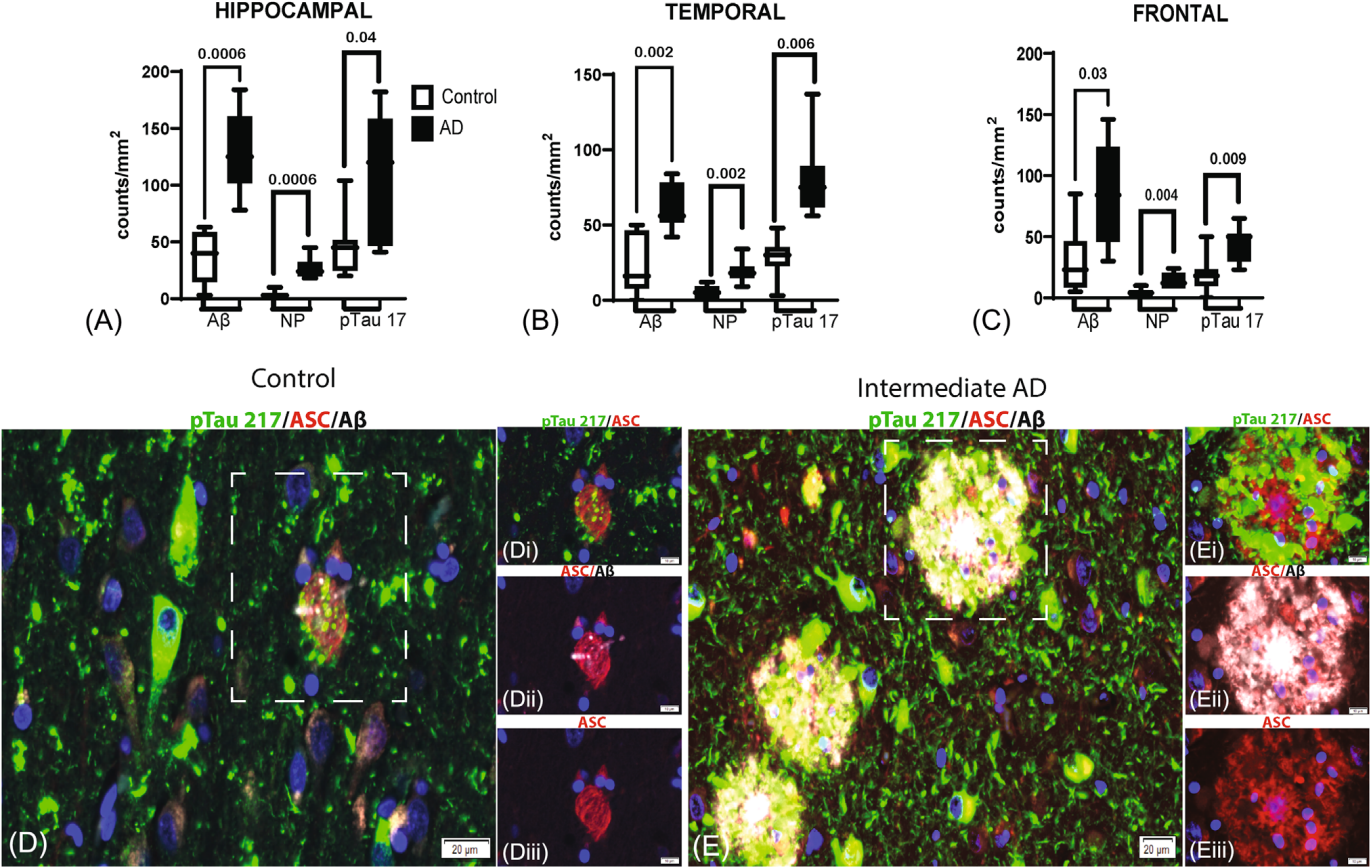
Protein expression of inflammasome complex seen in NPs. The number of A*β* plaques, NPs and pTau (Thr 217) NFTs differ between regions. (A)–(C) Significant increase in the number of A*β* plaques, NPs and NFTs in the intermediate AD cases compared to controls in all three regions. ASC (IC100; red; Diii) was seen in a few neurons (Di) in the controls (D) with minimal A*β* plaques (Dii). In intermediate AD cases (E), ASC (IC100; red; Eiii) was present in both NFTs and NPs as shown by A*β* (white) clusters with a core center (Eii) and pTau217 (green) dystrophic neurons (Ei). Beta Amyloid (A*β*); Neuritic Plaques (NP); pTau; Phosphorylated tau (pTau); Neurofibrillary tangles (NFTs); Alzheimer's disease (AD); Apoptosis‐associated Speck‐like protein containing a CARD (ASC).

Using a rabbit polyclonal antibody raised against pTau 217, a mouse monoclonal antibody raised against A*β*, and humanized monoclonal antibody raised against ASC/PYCARD (IC100), we were able to colocalize the inflammasome proteins detected in the NP in the ROIs. The NPs were defined as A*β* core and pTau217 positive dystrophic neurites and fragments. Our findings indicate that pTau217 immunoreactivity increased in the intermediate AD cases compared to controls in the hippocampus as well as the temporal and frontal lobes (Figure 4A–C; see Table 1). Since the hippocampus had presented the highest increase in NPs, we chose that region to detect ASC immunostaining in the A*β* core of the NP that colocalized with pTau217 positive neurons clustered in the NP core in the intermediate AD cases (Figure 4E,Ei–Eiii). In the controls, plaques were seen with A*β* and ASC, but these were more diffuse and did not colocalize (Figure 4D,Dii). In the CA1 region of the hippocampus, there were neurons that expressed pTau217, but only small neuritic threads were seen in the ASC positive clusters (Figure 4D,Di) in controls.

**TABLE 1:** Quantification of Aβ plaques, NPs, and NFTs.

Brain region	Counts (mm^2^)		Statistics
Hippocampal			
Control		AD	*p*‐value
A*β*	36 ± 22	130 ± 35	0.0006
NP	4 ± 3	28 ± 9	0.0006
pTau 217 tangle	47 ± 28	106 ± 59	0.04
Temporal			
	Control	AD	*p*‐value
A*β*	24 ± 19	61 ± 15	0.002
NP	5 ± 4	19 ± 7	0.002
pTau 217 tangle	28 ± 13	81 ± 27	0.0006
Frontal			
	Control	AD	*p*‐value
A*β*	33 ± 28	82 ± 42	0.03
NP	4 ± 3	14 ± 6	0.004
pTau 217 tangle	19 ± 16	81 ± 14	0.009

*Note*: Data are expressed as mean ± St. Dev.

Abbreviations: AD, Alzheimer's disease; A*β*, amyloid beta plaques; NP, neuritic plaques; NFT, neurofibrillary tangles (pTau 217).

**FIGURE 5:**
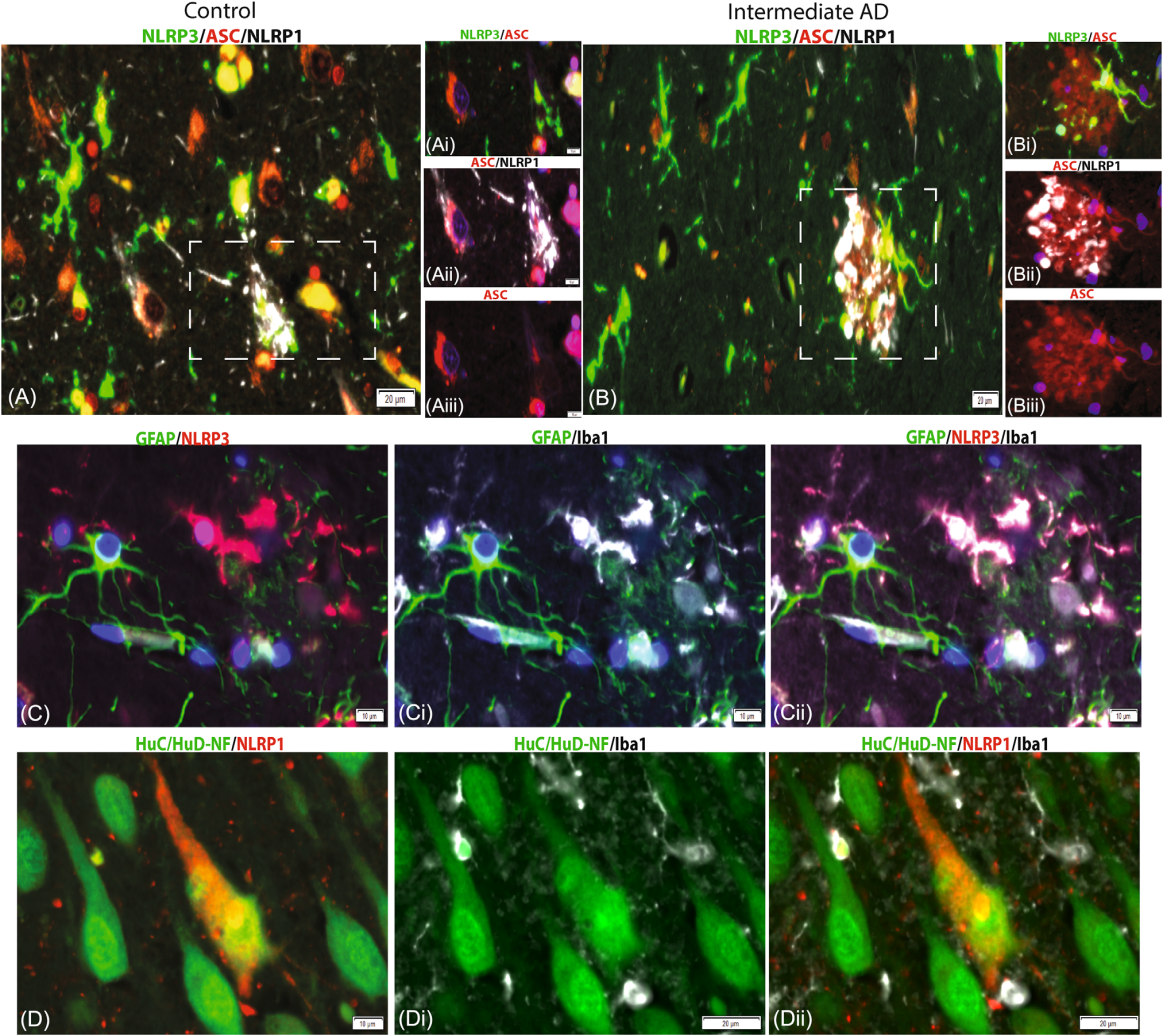
Inflammasome sensor proteins are seen in NPs. Protein expression of NLRP3 (green) is seen mainly in microglia‐shaped cells in both controls (A and Ai) and intermediate AD cases (B and Bi) that surround NLRP1 (white) and ASC (IC100; red; Aiii and Biii) positive neurons (Aii) and NPs (Bii). In (C), NLRP3 (red; C and Cii) is seen in the microglia‐shaped cells that colocalize with Iba1 (white; Ci and Cii), but not with GFAP (green). NLRP1 (red; D, Dii) expression is shown to overlap in the neuronal processes (green; D and Dii) but not in microglia (Di; white).NOD‐like receptor protein (NLRP); Alzheimer's disease (AD); Ionized calcium‐binding adaptor molecule 1 (Iba1); Glial Fibrillary Acidic Protein (GFAP).

In our previous experiments, we have shown that NLRP3 is detected mainly in microglia and NLRP1 is seen in neurons.^8^ Here, we sought to identify whether NLRP3 and NLRP1 were recruited in NPs in cases of intermediate AD. Using a mouse monoclonal antibody raised against NLRP3, a rabbit polyclonal NLRP1 antibody and IC100 we identified NLRP3 in microglial and NLRP1 in the NP and dendritic processes of neurons. In controls, NLRP3 was present in microglia that had fragments of ASC immunoreactivity (Figure 5A,Ai), whereas NLRP1 was detected in a few dendritic processes which colocalized with neuronal ASC (Figure 5A,Aii). In intermediate AD cases, IC100 immunoreactivity was present in the NP, which showed dense expression of NLRP1 (Figure 5B,Bii). Surrounding the NPs were NLRP3 positive microglia‐like structures, which presented central ASC colocalization (Figure 5B,Bi). We further confirmed that the NLRP3 expression was in microglia and not astrocytes using a chicken polyclonal antibody raised against Iba1 and a mouse monoclonal antibody raised against GFAP. The results showed that the NLRP3 protein expression was in Iba1 positive microglia and not in GFAP positive astrocytes (Figure 5C,Cii). We used a cocktail of mouse anti‐HuC/HuD and mouse anti‐NF antibodies mixed with the rabbit‐NLRP1 antibody and the chicken‐Iba1 antibodies to determine if the NLRP1 protein is mainly neuronal dendrites. NLRP1 was seen in the neuronal processes defined by the HuC/HuD and NF expression and was not detected in Iba1 (Figure 5D,Dii).

**FIGURE 6:**
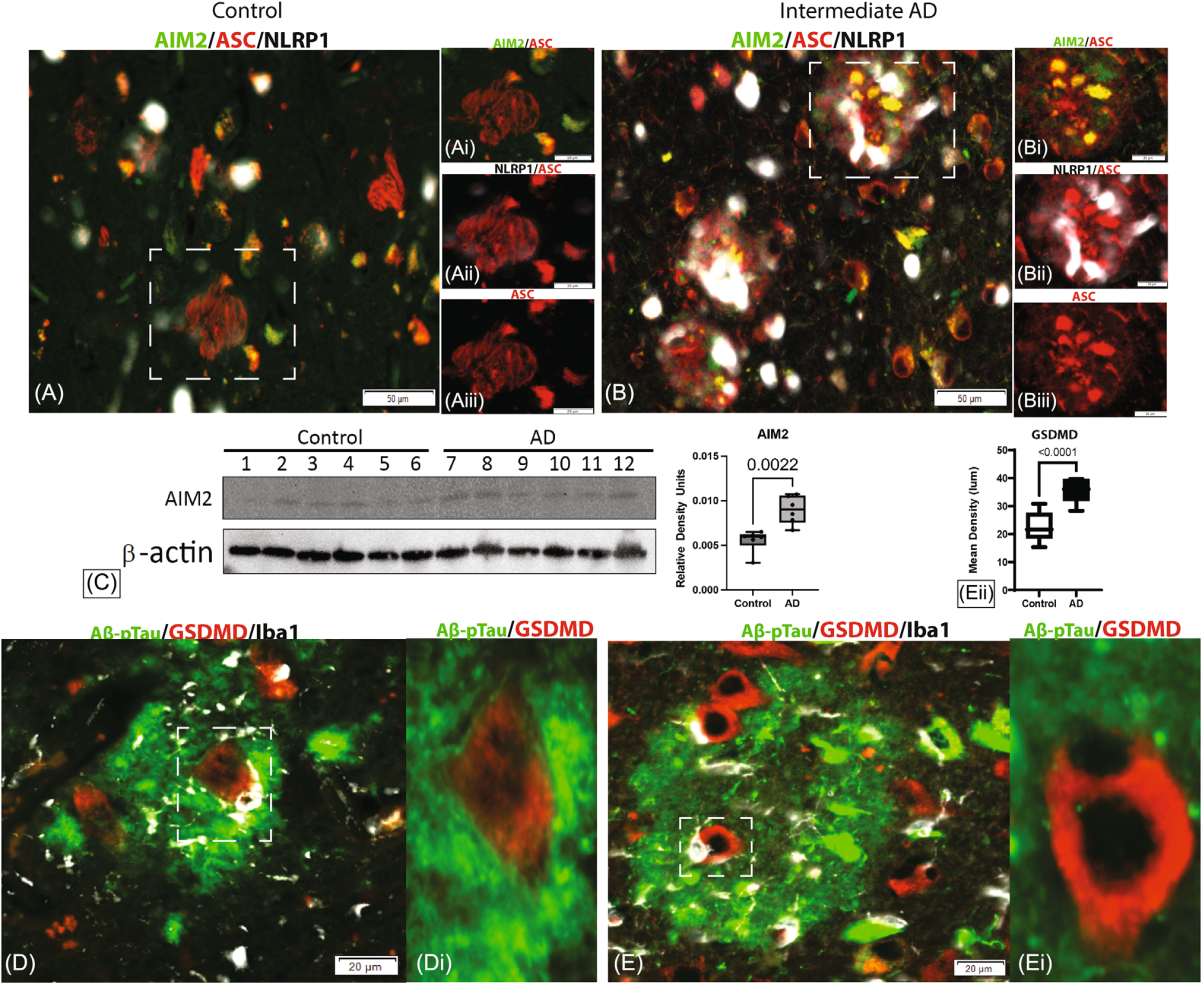
AIM2 and GSDMD expression increases in intermediate AD. (A) Punctate AIM2 expression (green) is seen in controls (Ai), and a pronounced accumulation of AIM2 in the NP is seen in the intermediate AD case (B, Bi). In the clusters of ASC (A, Aiii, B and BiiI), AIM2 did not colocalize with NLRP1 (white) in either controls (Aii) or intermediate AD (Bii) cases. (C) Immunoblot analysis of AIM2 showing AIM2 protein expression is significantly higher in the intermediate AD cases compared to controls. Sparse amounts of GSDMD expression is seen in neurons (red) near diffuse A*β* plaques (green) in controls (D and Di). In the intermediate AD cases, the dense A*β* plaque formation with NFTs shows multiple neuronal cells with dense GSDMD signaling (E and Ei) and surrounding microglia (white; E). Luminosity density analysis of GSDMD showing GSDMD immunofluorescence is significantly higher in the intermediate AD cases compared to controls (Eii). Neuritic Plaques (NP); NOD‐like receptor protein (NLRP); Apoptosis‐associated Speck‐like protein containing a CARD (ASC); Alzheimer's disease (AD); Absent in Melanoma 2 (AIM2). Amyloid (A*β*); Neurofibrillary tangles (NFTs); Alzheimer's disease (AD).

We investigated whether the sensor protein AIM2 was evident in NPs. In controls, AIM2 expression was sporadic, and it did not colocalize with the fragments of the filamentous ASC positive neurons (Figure 6A,Ai). Moreover, there was an overlap of AIM2 and ASC in some minute dense ASC positive regions, but there was no colocalization with NLRP1 (Figure 6A,Aii). In the intermediate AD cases, in the NP, AIM2 immunoreactivity was more prevalent and colocalized with fragments of ASC positive clusters (Figure 6B,Bi). Dense NLRP1 immunoreactivity was also present in the NP and colocalized to parts of the ASC cluster in fragments adjacent to AIM2‐ASC positive structures, but these three proteins did not colocalize (Figure 6B,Bii). In addition, the immunoblot analysis showed a significant increase in the protein expression of AIM2; *p* = 0.002 (Figure 6C).

Finally, we investigated if the upregulation of inflammasome sensors seen in these series of experiments showed an effect of triggering a downstream mediated pyroptosis response in the presence of AD morphological changes. Using triple‐labeling IF for A*β*/pTau (AT8), GSDMD, and Iba1, we investigated if GSDMD protein could be detected with AD neuropathological changes. The neurons detected adjacent to diffuse A*β* plaques seen in the control group showed sparse GSDMD protein expression (Figure 6D,Di). In proximity to the A*β* plaques and NFTs seen in the intermediate group, there was a prominent expression of GSDMD protein around the cell membrane of neurons with pools of Iba1 positive microglia (Figure 6E,Ei). The mean luminosity density analysis of GSDMD immunoreactivity was significantly increased in neuronal cell bodies in the intermediate AD group compared to the control; *p* = <0.0001 (Figure 6Eii).

### 3.3 Pathway activation

**FIGURE 7:**
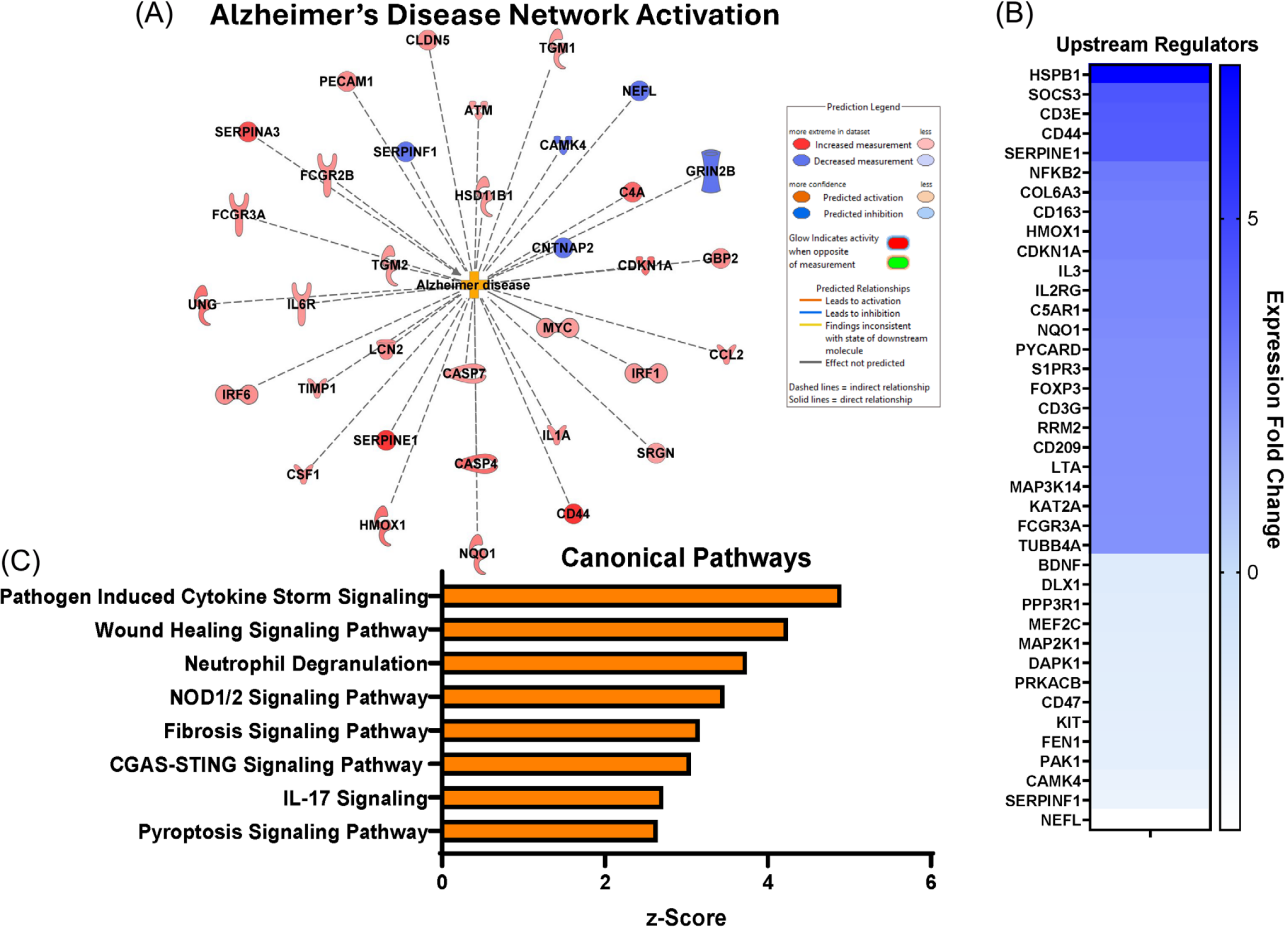
AD network activation. (A) IPA‐predicted activation of Alzheimer's disease (AD) Network shows several pro‐inflammatory genes upregulated. (B) Analysis of upstream regulators of the AD Network revealed several enriched pro‐inflammatory genes that include the inflammasome adapter protein *PYCARD*. (C) Canonical pathway analysis showed activation of several pro‐inflammatory pathways that include the pyroptosis signaling pathway.

AD pathology was more prominent in the temporal lobe than what is seen in the frontal lobe in the intermediate AD cases (Figure 4). In the hippocampus, we showed an increase in inflammatory genes and a decrease in neuronal plasticity genes at the intermediate AD stage compared to controls. It is common for controls to show age‐related changes with a varying degree of NFTs and A*β* plaques in certain hippocampal structures.^8^ Thus, we used mRNA counts in the temporal lobe to investigate AD network activation trends. IPA demonstrated activation of the AD network with several pro‐inflammatory genes that include *IL6R*, *IL1A*, and *CCL2* to be among the top enriched genes (Figure 7A). In addition, several upstream regulators were found to be highly enriched that include the inflammasome adaptor, *PYCARD*, along with *CD163*, supporting a role for T cells and macrophages in AD pathology (Figure 7B). Moreover, we identified several canonical pathways to be highly activated that include the Pyroptosis Signaling Pathway, further supporting a role for inflammasomes in AD pathology (Figure 7C). We further assessed the role of the Pyroptosis Signaling pathway in the development of AD. IPA demonstrated significant expression of multiple inflammasomes that include *AIM2*, *NLRC4*, and *NLRP3*. In addition, we found activation of the TLR/NFκB pathway leading to induction of pro‐inflammatory and inflammasome gene expression. Furthermore, these findings support the hypothesis that inflammasome‐driven transcriptional networks are deregulated and that they contribute to AD pathology (Supplementary Data 5).

## 4 DISCUSSION

In this study, we investigated the mRNA expression of 758 genes in the hippocampus, temporal lobe, and in the frontal lobe in postmortem cases of intermediate AD and controls. Furthermore, we compared the sex differences seen in females and males with intermediate AD and compared them to same‐sex controls. In addition, we analyzed the expression pattern of inflammatory genes and inflammasome proteins in the ROI and show that the expression of inflammasome mRNA is associated with protein expression of *TREM2*, *ASC*, *NLRP1*, *NLRP3*, *caspases‐1*, *‐8*, and *AIM2* that may be involved in inflammasome signaling. Our data findings indicate that hippocampal expression of inflammasome genes is significantly increased compared to temporal and frontal regions, consistent with the clinical findings of MCI often described in donors that have intermediate AD neuropathological findings.

Our results demonstrate that in the intermediate stage of AD, *APOE* gene expression is upregulated in the intermediate AD cases compared to controls in the hippocampus but not in the temporal and frontal lobe. Of interest, we found that in females with intermediate AD pathology, the upregulation of *APOE* and the downregulation of *NRGN* genes were significantly changed in both the hippocampal and temporal regions. Recently, it has been shown that *APOE* inactivation in the hippocampus interferes with adult neurogenesis and downregulates synaptic function while promoting inflammatory responses.^47^, ^48^ In addition, NRGN, a protein important for postsynaptic transmission, is downregulated in the hippocampus, temporal and frontal lobes. NRGN is important to maintain neuronal plasticity and synaptic function; others have shown that NRGN is lost when tauopathies increase in AD.^49^, ^50^ The reduction of *NRGN* mRNA seen in this study supports findings of other studies, which demonstrate that changes in NRGN expression are associated with memory loss and decreases in executive function.^51^ One study showed that NGRN protein expression is significantly decreased in postmortem samples using AD cases with high AD level neuropathological changes across multiple brain regions with no difference between females and male donors.^52^ However, a more recent study has shown that females have a higher level of NRGN in the CSF, which also correlated with higher levels of A*β* positron emission tomography uptake.^53^ Here we show that at the stage of intermediate AD, there are alterations to synaptic plasticity genes which may be induced prior to inflammatory mediators and warrant further investigations.

We have previously shown that ASC is elevated in the serum of individuals with MCI.^7^ Aligned with region specific tauopathies in AD, our recent study has shown that in the intermediate stage of AD (i.e., Braak NFT stage, III–IV) an increase expression of inflammasome proteins (ASC) correlate with the spread of NFTs seen in regions of the hippocampus.^8^ Moreover, using a humanized monoclonal anti‐ASC antibody raised against the PYRIN domain (PYD), IC100, we noted expression in neurons with dystrophic neurites, whereas, using a monoclonal commercially available ASC antibody, we detected expression in microglia. Moreover, we reported an increase in the inflammasome sensor proteins NLRP1 and NLRP3 in hippocampal neurons and microglia in postmortem tissue‐sections with intermediate AD neuropathology.^8^ In addition, we have shown that in the intermediate stage of AD, a decrease in post‐synaptic proteins is associated with the spread of NFTs and ASC specks when comparing donors with age‐related changes (low AD) to donors with intermediate AD neuropathological changes.^50^

In the ROIs, we found a 20%–38% upregulation of the genes included in the mRNA profiling panels that we investigated. Of those genes that were upregulated, 58% were considered to be associated with inflammatory processes. As expected, the hippocampus ranked the highest in inflammatory gene responses (42%), whereas the temporal lobe ranked a bit lower (36%) and the frontal lobe was lowest (20%) in the regions studied. *TREM2* is thought to regulate microglia receptors and enhance phagocytic properties to clear A*β* plaques and regulate the inflammatory responses.^12^ Dysregulation of TREM2 has been shown to impair microglia phagocytic function resulting in a higher NLRP3 response.^20^ In the regions that we investigated we did not see a change in *TREM2* gene expression when females and males with intermediate AD cases were pooled together, which may relate to a dysfunctional TREM2 response and in turn there was an increase in inflammasome signaling.

Interestingly, there were regional differences in apoptotic‐related caspase genes thought to initiate inflammatory responses. For instance, caspase‐4 was lower in the hippocampus than in the temporal and frontal lobes. Caspase‐4 has been shown to play a critical role in inflammatory cell death and primes NLRP3 and caspase‐1 activation.^54^ In addition, we show a significant change in caspase‐9 in the hippocampal than what was seen in the temporal and frontal lobes. In all of the ROIs we found an increase in caspase‐6 in the intermediate AD cases. Active caspase‐6 has been shown to be present in neurons prior to apoptotic activity and can induce axonal degeneration, which has been correlated with MCI.^55^ Caspase‐6 acts by cleaving tau proteins, increasing the levels of A*β* peptide production and facilitating the assembly of NLRP3.^39^, ^55^, ^56^ Here we show that caspases play a pivotal role in intermediate AD pathology and coincide with inflammasome formation.

These findings demonstrate that caspase RNA expression is associated with the propagation of Tau and A*β* deposition.^57^ However, our findings did not show a significant upregulation of caspase‐3 in intermediate AD cases, suggesting that apoptosis may not play a role at this stage of AD pathology. Others have shown an increase in caspase‐3 in neurons, astrocytes, and blood vessels in high AD neuropathological stage, which is indicative of neuronal degeneration, gliosis, and a decrease in post‐synaptic densities.^58^, ^59^ Further studies using cases with high AD staging may be needed to show an increase in caspase‐3‐induced apoptosis forms across brain regions.

The RNA counts for the genes involved in the inflammasome complex showed a significant upregulation in *caspase‐1*, *ASC/PYCARD*, *NLRP1*, *NLRP3*, and *IL‐18* in the hippocampus in intermediate AD cases compared to controls. Additionally, we investigated noncanonical inflammasome pathway activation of caspase‐8, which increased in the hippocampal samples of the intermediate AD cases. The temporal area showed an upregulation of *caspase‐1* and *PYCARD*, but the frontal area only had an upregulation of *caspase‐1* RNA in the intermediate AD cases. These findings suggest that the propagation of tauopathies and A*β* deposition seen in AD neuropathology is associated with a region‐specific inflammatory mediation,^24^, ^26^ but there may be a response to various pathological stimuli that triggers caspase‐1 production prior to inflammation^60^ and by other pathways.^15^, ^57^

When we controlled for sex differences, we found that females with intermediate AD had significant upregulation of inflammasome genes (NLRs, *PYCARD*, and *Caspase‐1*) in the hippocampal and temporal regions. The males showed upregulation of the inflammasome genes in the hippocampal region but only showed an upregulation of the *caspase‐1* gene in the temporal and frontal regions. The data supports the findings of Cyr and Rivero Vaccari (2023), who showed that inflammasome proteins are increased in aged female mice compared to aged males.^61^ In human studies that investigate sex differences in patients, AD has been found that the cognitive decline occurs faster in women compared to men, which may indicate that AD biomarkers and therapies may need to investigate the difference in risk factor profiles between men and women.^34^, ^53^

Here we investigated the neuropathology and inflammasome protein expression seen in AD associated NP formation. Isocortical NFTs and NP propagation are correlated with increased dementia scores detected with the mini‐mental state examination completed prior to death.^62^ At the intermediate AD stage there was a higher density of NP detected in the hippocampus. Recently, we showed an increase in NLRP1 and NLRP3 in intermediate AD hippocampal sections in neurons and microglia.^8^ In addition, we demonstrated that ASC (IC100) protein could be detected mainly in neurons that expressed NLRP1, and caspase‐1 was seen in the extracellular matrix in cases with intermediate AD in the hippocampus sections.^8^ In these series of experiments, we found that ASC is present within the NP formation along with pTau217 dystrophic neurons and A*β* proteins. In addition, we show that NLRP1 is present in NP clusters and NLRP3‐positive microglia can be seen adjacent to NP formation. Other studies have shown that NLRP1 can activate the inflammasome via activation of caspase‐1 and induction of IL‐1*β* and IL‐18, which initiates an innate immune response.^6^ Furthermore, studies have shown that NLRP3 is upregulated A*β* aggerates and tau hyperphosphorylation, both of which activates inflammasomes in microglial cells.^39^

Finally, we sought to determine whether the NP sites contain dystrophic neurites surrounded by A*β* deposits show increased inflammasome gene and protein expression. In the hippocampus, we found an increase in NP protein structures and an upregulation of *IL‐18* genes in the intermediate AD cases. The release of damage associated molecular patterns (DAMPs) in the form of self‐dsDNA released from cells surrounding the NP is thought to activate the innate immune system.^63^ It is possible that in response to DAMPs released in the NP, AIM2 forms an inflammasome with ASC, supporting our earlier findings (10). AIM2 contains a C‐terminal HIN‐200 domain that binds to the ds‐DNA in the cytoplasm and an N‐terminal that binds to the Pyrin domain (PYD) of ASC.^13^, ^63^ The formation of the AIM2 inflammasome complex attracts pro‐caspase‐1 molecules in close proximity to each other, resulting in the release of active caspase‐1 and the induction of IL‐1*β* and IL‐18 cytokines and pyroptotic cell death.^14^

In the intermediate AD cases, AIM2 protein expression was upregulated as determined by immunoblotting immunohistochemistry combined with microscopy procedures. In controls, we found sporadic clusters of AIM2 expression that were adjacent to ASC filamentous structures. In intermediate AD cases, AIM2 was clustered in the NPs and colocalized with a dense expression of ASC. Although NLRP1 immunoreactivity did not overlap with AIM2, these proteins seemed to be adjacent to one another and bound to ASC. The other sensor proteins, NLRP1 and NLRP3, were also present in the NPs in intermediate AD cases, although the two proteins did not overlap, but each protein did colocalize with ASC. NLRP1 was seen within the cluster, whereas NLRP3 was found mainly on the microglia present captured in the NP. Moreover, we found that GSDMD immunoreactivity is more prevalent in neuronal cell bodies that are adjacent and involved in NFTs and A*β* plaques, indicating that pyroptotic mechanisms drive neuronal death in AD pathology.

In conclusion, our findings indicate that in intermediate AD neuropathological change, there is region‐specific upregulation of inflammasome signaling, which is intensified in females. In addition, we found that ASC is present in pTau217 positive neurons and NPs recognized by a humanized antibody raised against the PYD domain which supports the theory that inflammasomes are potential therapeutic targets to delay dementia related diseases. Further investigations that include high AD neuropathological groups may identify other inflammasome sensors besides AIM2, which may exacerbate the inflammatory response. A limitation of postmortem studies is that there is only one timepoint. Thus, more investigations using antemortem plasma and CSF at various clinical stages of AD related dementia would be beneficial to comprehending the pathophysiology of inflammasomes in AD.

## 5 AUTHOR CONTRIBUTIONS

JPdRV contributed to western blot analyses and pathwayanalyses. DAD prepared the tissue samples for the gene analysis. APScontributed to pathway analysis and interpretation. SPG prepared the RNA fromthe tissue samples. XS and AB assisted in the clinical evaluation of thedonors. SHG carried out the neuropathological evaluations for the donors. WDD, HMB and RWK evaluated inflammasome interpretation. RTV preformed the geneanalyses and did the immunohistology imaging. All authors were involved in thepreparation and final editing of the manuscript.

## FUNDING INFORMATION

This research was funded by a grant from the University of Miami and the State of Florida, Department of Health (COHFA) awarded to WDD.

## CONFLICT OF INTEREST STATEMENT

JPdRV, HMB, RWK, WDD, and RVT are co‐founders and managing members of InflamaCORE, LLC and have licensed patents on inflammasome proteins as biomarkers of injury and disease as well as on targeting inflammasome proteins for therapeutic purposes. JPdRV, HMB, RWK, and WDD are Scientific Advisory Board Members of ZyVersa Therapeutics. DAD declares no conflicts of interest. APW declares no conflicts of interest. SPG declares no conflicts of interest. XS declares no conflicts of interest. AB declares no conflicts of interest. SHG declares no conflicts of interest.

## Supporting information


**Supplementary Data 1.** Donor demographics.

**Supplementary Data 2.** Hippocampal sex differences in mRNA readings.

**Supplementary Data 3.** Temporal sex differences in mRNA readings.

**Supplementary Data 4.** Frontal sex differences in mRNA readings.

**Supplementary Data 5.** Inflammasome‐driven transcriptional networks seen in the temporal lobe.

## Data Availability

The data that supports the findings of this study are available in the supplementary material of this article.
