# Supplementary material for: Increased expression of inflammasome signaling genes and proteins in selective brain regions in the intermediate stage of Alzheimer's disease
Source: Brain Pathol. 2026 Feb 22;36(5):e70086. doi: 10.1111/bpa.70086 (PMC13429301; doi:10.1111/bpa.70086)
Supplement: Supplementary file 1 — Supplementary Data 1. Donor demographics. [file BPA-36-e70086-s005.pdf]

Supplementary Data 1.										
Case Demographics										
	Age	Sex	PMI	A	C	B	Braak	Clinical Assessment	COD	RACE
Control										
1	88	F	9.00	1	1	1	Stage I	No Cognitive Disorder	Arteriovascular sclerotic disease	Caucasian
2	87	M	8.00	1	0	1	Stage I	No Cognitive Disorder	Cardiac Arrest;Atherosclerotic Disease	Caucasian
3	86	F	37.00	1	0	1	Stage II	No Cognitive Disorder	urethral cancer, heart murmur	Caucasian
4	89	F	4.55	1	1	1	Stage II	No Cognitive Disorder	Coronary Artery Disease;Atrial fibrillation;Hypertension	Caucasian
5	96	F	6.00	0	1	1	Stage II	No Cognitive Disorder	Generalized atherosclerosis	Caucasian
6	72	M	17.25	0	0	1	Stage I	No Cognitive Disorder	Atherosclerotic Coronary Artery Disease , Ischemic Cardiomyopathy	Caucasian
7	72	M	17.66	0	0	0	Stage 0	No Cognitive Disorder	Acute Myocardial Infarction;Diabetes Mellitus	Caucasian
8	73	M	20.50	2	0	1	Stage I	No Cognitive Disorder	Atherosclerotic Heart Disease, Cardiomyopathy, Diabetes	Caucasian
9	67	F	25.95	1	0	1	Stage I	No Cognitive Disorder	Atherosclerotic Coronary Artery Disease, Tobacco Abuse, Hypertension	Caucasian
10	94	M	23.40	2	0	1	Stage I	No Cognitive Disorder	Congestive Heart Failure	Caucasian
11	90	F	24.30	1	1	1	Stage II	No Cognitive Disorder	Cardiac Arrest;Respiratory Arrest;Unspecified Natural Causes;Cardiovascular Diseae	Caucasian
12	93	M	16.88	1	1	1	Stage II	No Cognitive Disorder	Cardiac Arrest;Atherosclerotic Disease	Caucasian
Mean	84		17.54							
Intermediate AD										
1	92	M	12.00	3	3	2	Stage IV	Moderate	Acute Renal Failure	Caucasian
2	90	F	8.48	2	3	2	Stage III	MCI	Acute Renal Failure	Caucasian
3	90	F	22.12	2	3	2	Stage IV	Severe	Advanced Dementia;vascular	Caucasian
4	91	F	15.00	3	2	2	Stage III	MCI	Lung cancer	Caucasian
5	79	F	18.28	2	3	2	Stage III	Moderate	Cardiac Arrest;Atherosclerotic Disease	Caucasian
6	82	M	17.60	2	3	2	Stage IV	Moderate	Cardiac Arrest;Atherosclerotic Disease	Caucasian
7	87	M	13.91	2	3	3	Stage V	Moderate	Sepsis due to urinary tract infection	Caucasian
8	78	M	14.51	2	2	2	Stage III	Moderate	Cardiac Arrest;Atherosclerotic Disease	Caucasian
9	78	F	6.00	2	2	2	Stage III	Moderate	Cardiac Arrest;Atherosclerotic Disease	Caucasian
10	83	M	15.12	2	2	2	Stage IV	Moderate	Liver Failure	Caucasian
11	77	F	10.75	2	2	2	Stage III	Moderate	Cardiac Arrest;Atherosclerotic Disease	Caucasian
12	90	F	13.25	2	1	2	Stage IV	Moderate	Lung Cancer	Caucasian
Mean	85		13.92							

PMI=postmortem interval; Yr=year; A Score=Aβ immunopositivity, Thal Phase; C Score=neuritic plaque density, CERAD; B Score=Neurofibrillary tangles; COD= Cause of Death; AD= Alzheimer's disease; M = male; F = female; No Cognitive Disorder ≥ 62 % on Modified Telephone Interview for Cognitive Status (TICS-M) score; MCI=Mild Cognitive Impairment (TICS-M score 61-54%); Moderate=Moderate dementia (TICS-M score 5336%); Severe=Severe dementia ; Case data adapted from published in Vontell, Regina T et al. “Identification of inflammasome signaling proteins in neurons and microglia in early and intermediate stages of Alzheimer's disease.” *Brain pathology*, vol. 33,4 (2023): e13142. doi:10.1111/bpa.13142

Supplementary data. Table 2 mRNA Data

Hippocampal				Temporal				Frontal		
	p value	FDR			p value	FDR			p value	FDR
APOE	0.00876	0.019		*APOE*	0.189519	0.21		*APOE*	0.552739	0.65
CASP1	0.043416	0.050		*CASP1*	0.000681	0.01		*CASP1*	0.01822	0.05
CASP3	0.912709	0.776		*CASP3*	0.085761	0.12		*CASP3*	0.909399	0.97
CASP4	0.016539	0.029		*CASP4*	0.000121	0.01		*CASP4*	0.0000907	0.01
CASP6	0.002717	0.010		*CASP6*	0.005395	0.02		*CASP6*	0.008353	0.05
CASP8	0.000094	0.002		*CASP8*	0.066793	0.10		*CASP8*	0.391369	0.53
CASP9	0.048869	0.050		*CASP9*	0.05619	0.09		*CASP9*	0.162852	0.31
IL18	0.000129	0.002		*IL18*	0.394998	0.44		*IL18*	0.211927	0.49
NLRP1	0.005704	0.014		*NLRP1*	0.178866	0.20		*NLRP1*	0.348078	0.64
NLRP3	0.001338	0.007		*NLRP3*	0.933394	0.95		*NLRP3*	0.315514	0.60
NRGN	0.0085	0.034		*NRGN*	0.029908	0.05		*NRGN*	0.017787	0.05
PYCARD	6.75E-05	0.001		*PYCARD*	0.00428	0.02		*PYCARD*	0.111702	0.25
TREM2	0.0553	0.064		*TREM2*	0.100623	0.30		*TREM2*	0.2568875	0.54
