# Supplementary material for: Increased expression of inflammasome signaling genes and proteins in selective brain regions in the intermediate stage of Alzheimer's disease
Source: Brain Pathol. 2026 Feb 22;36(5):e70086. doi: 10.1111/bpa.70086 (PMC13429301; doi:10.1111/bpa.70086)
Supplement: Supplementary file 2 — Supplementary Data 2. Hippocampal sex differences in mRNA readings. [file BPA-36-e70086-s001.pdf]

Supplementary Figure 2

Hippocampal

A

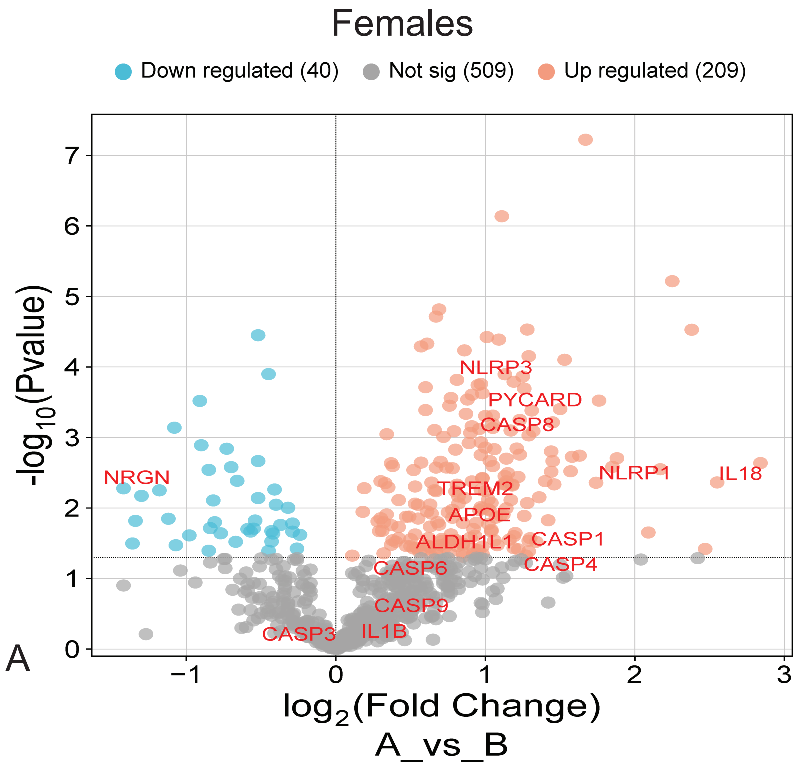

B

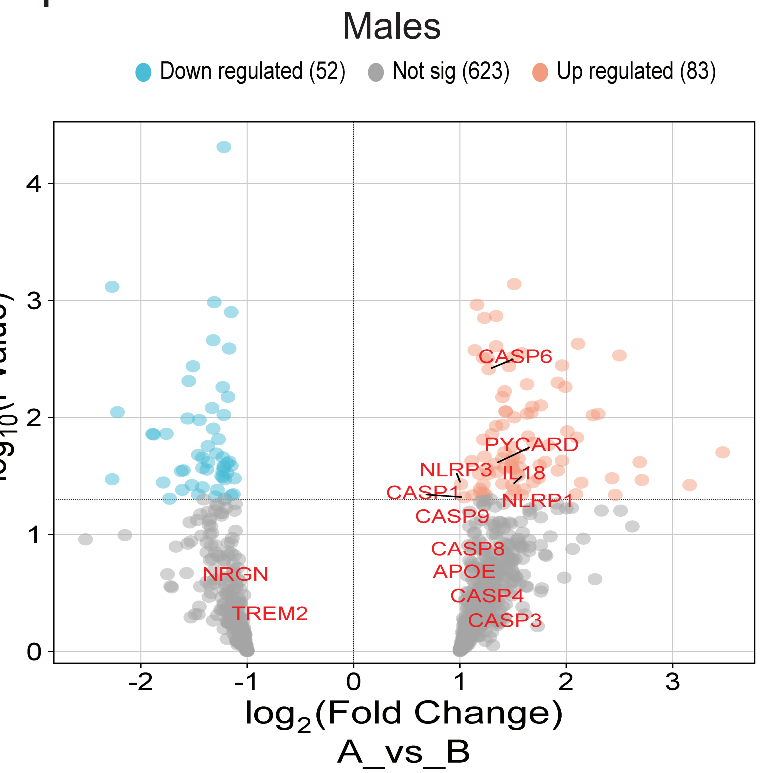

C

Hippocampal						
	**Females**				**Males**	
	**p value**	**FDR**			**p value**	**FDR**
APOE	**0.04**	**0.05**		***APOE***	**0.18**	**0.21**
CASP1	**0.04**	**0.05**		***CASP1***	**0.04**	**0.05**
CASP3	**0.92**	**0.98**		***CASP3***	**0.60**	**0.70**
CASP4	**0.06**	**0.07**		***CASP4***	**0.28**	**0.50**
CASP6	**0.44**	**0.68**		***CASP6***	**0.00**	**0.01**
CASP8	**0.00**	**0.00**		***CASP8***	**0.12**	**0.25**
CASP9	**0.31**	**0.35**		***CASP9***	**0.08**	**0.15**
IL18	**0.03**	**0.04**		***IL18***	**0.04**	**0.05**
NLRP1	**0.03**	**0.04**		***NLRP1***	**0.04**	**0.05**
NLRP3	**0.00**	**0.00**		***NLRP3***	**0.03**	**0.04**
NRGN	**0.01**	**0.03**		***NRGN***	**0.27**	**0.30**
PYCARD	**0.00**	**0.00**		***PYCARD***	**0.02**	**0.04**
TREM2	**0.01**	**0.03**		***TREM2***	**0.57**	**0.63**
