# Supplementary material for: Increased expression of inflammasome signaling genes and proteins in selective brain regions in the intermediate stage of Alzheimer's disease
Source: Brain Pathol. 2026 Feb 22;36(5):e70086. doi: 10.1111/bpa.70086 (PMC13429301; doi:10.1111/bpa.70086)
Supplement: Supplementary file 3 — Supplementary Data 3. Temporal sex differences in mRNA readings. [file BPA-36-e70086-s002.pdf]

Supplementary Figure 3

Temporal

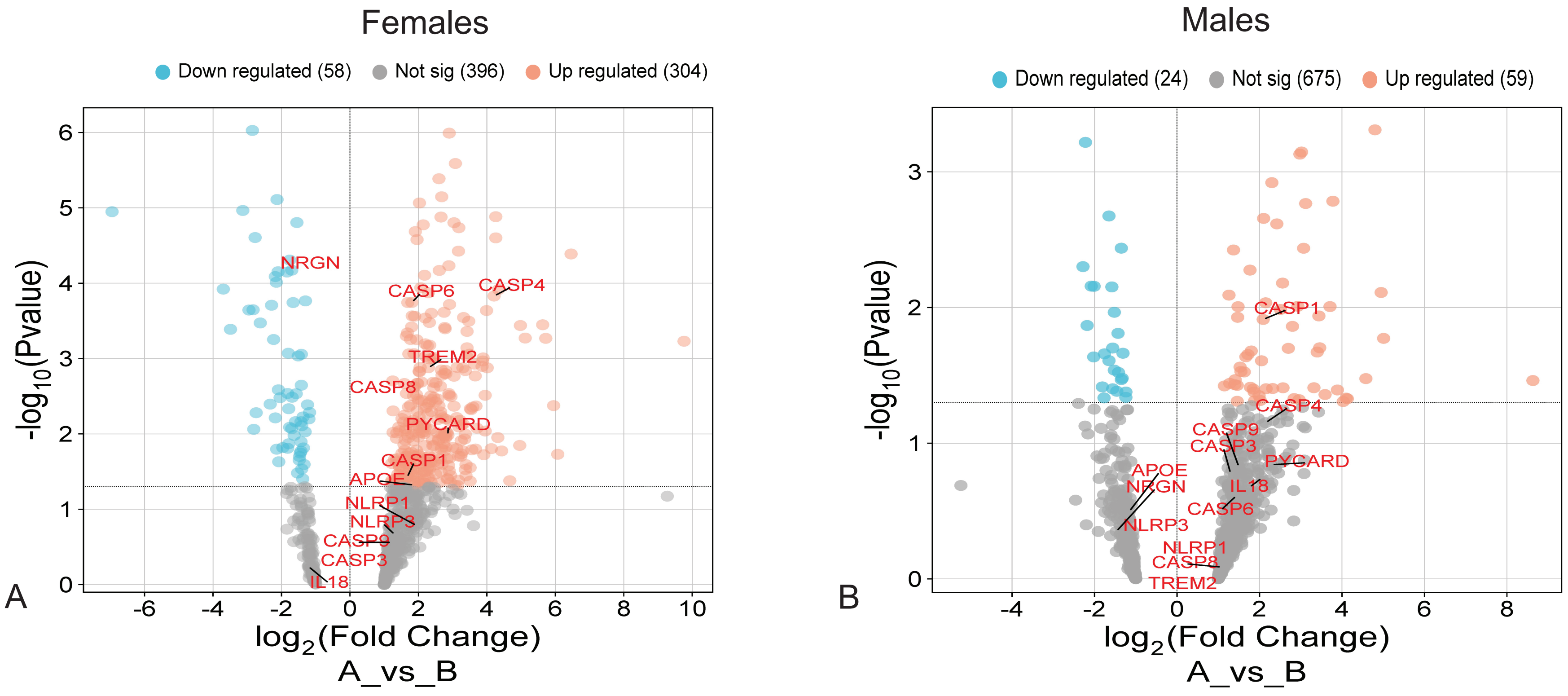

C

Temporal						
Females				Males		
	p value	FDR			p value	FDR
APOE	**0.04**	**0.05**		***APOE***	**0.32**	**0.84**
CASP1	**0.04**	**0.05**		***CASP1***	**0.01**	**0.03**
CASP3	**0.40**	**0.58**		***CASP3***	**0.17**	**0.70**
CASP4	**0.00**	**0.00**		***CASP4***	**0.07**	**0.09**
CASP6	**0.00**	**0.00**		***CASP6***	**0.24**	**0.50**
CASP8	**0.00**	**0.00**		***CASP8***	**0.82**	**0.95**
CASP9	**0.27**	**0.55**		***CASP9***	**0.15**	**0.46**
IL18	**0.57**	**0.60**		***IL18***	**0.18**	**0.35**
NLRP1	**0.17**	**0.40**		***NLRP1***	**0.68**	**0.90**
NLRP3	**0.22**	**0.50**		***NLRP3***	**0.46**	**0.80**
NRGN	**0.00**	**0.00**		***NRGN***	**0.45**	**0.85**
PYCARD	**0.01**	**0.02**		***PYCARD***	**0.14**	**0.70**
TREM2	**0.00**	**0.01**		***TREM2***	**0.94**	**1.00**
