# Supplementary material for: Increased expression of inflammasome signaling genes and proteins in selective brain regions in the intermediate stage of Alzheimer's disease
Source: Brain Pathol. 2026 Feb 22;36(5):e70086. doi: 10.1111/bpa.70086 (PMC13429301; doi:10.1111/bpa.70086)
Supplement: Supplementary file 4 — Supplementary Data 4. Frontal sex differences in mRNA readings. [file BPA-36-e70086-s004.pdf]

Supplementary Figure 4

Frontal

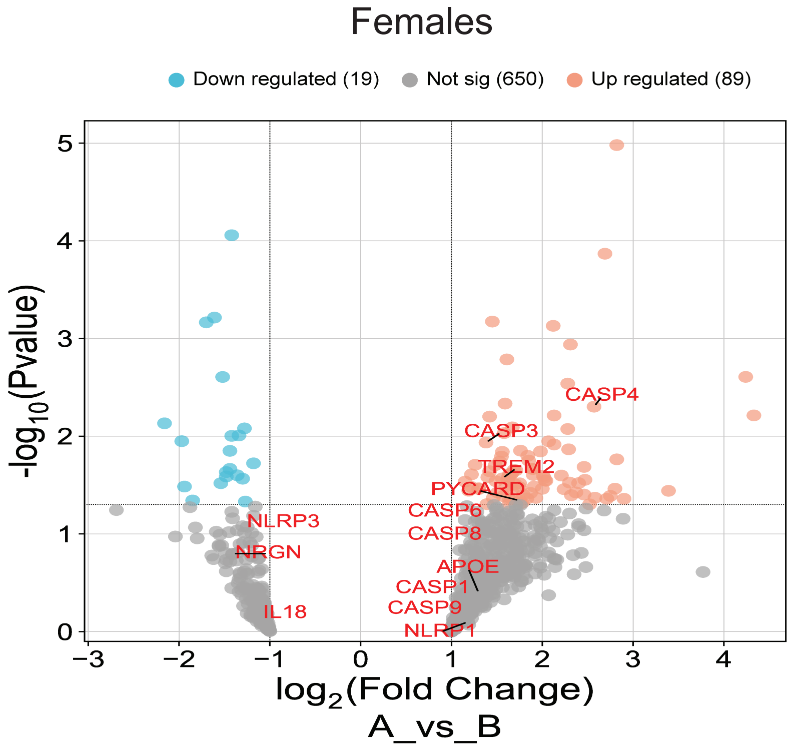

A

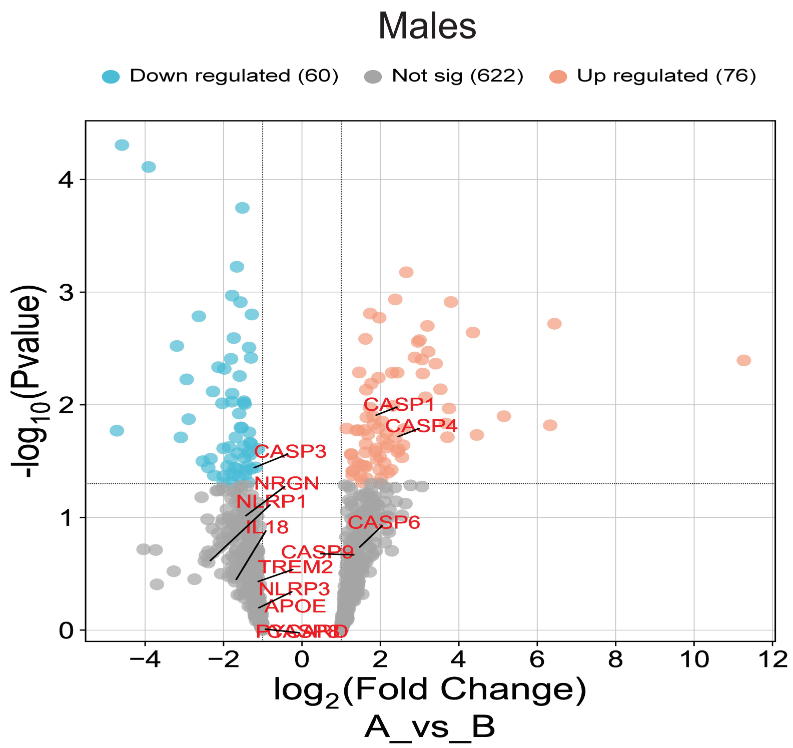

B

Frontal						
Females				Males		
	p value	FDR			p value	FDR
APOE	0.40	0.85		*APOE*	0.78	0.99
CASP1	0.47	0.90		*CASP1*	0.01	0.05
CASP3	0.01	0.04		*CASP3*	0.04	0.05
CASP4	0.01	0.01		*CASP4*	0.02	0.05
CASP6	0.08	0.09		*CASP6*	0.19	0.21
CASP8	0.13	0.25		*CASP8*	0.90	1.00
CASP9	0.48	0.91		*CASP9*	0.22	0.27
IL18	0.77	1.00		*IL18*	0.37	0.58
NLRP1	0.79	1.00		*NLRP1*	0.25	0.35
NLRP3	0.10	0.25		*NLRP3*	0.65	0.91
NRGN	0.16	0.60		*NRGN*	0.10	0.29
PYCARD	0.05	0.05		*PYCARD*	0.97	1.00
TREM2	0.03	0.04		*TREM2*	0.38	0.69

C
