# Supplementary figures and images for: Increased expression of inflammasome signaling genes and proteins in selective brain regions in the intermediate stage of Alzheimer's disease
Source: Brain Pathol. 2026 Feb 22;36(5):e70086. doi: 10.1111/bpa.70086 (PMC13429301; doi:10.1111/bpa.70086)

Supplementary Figure 5

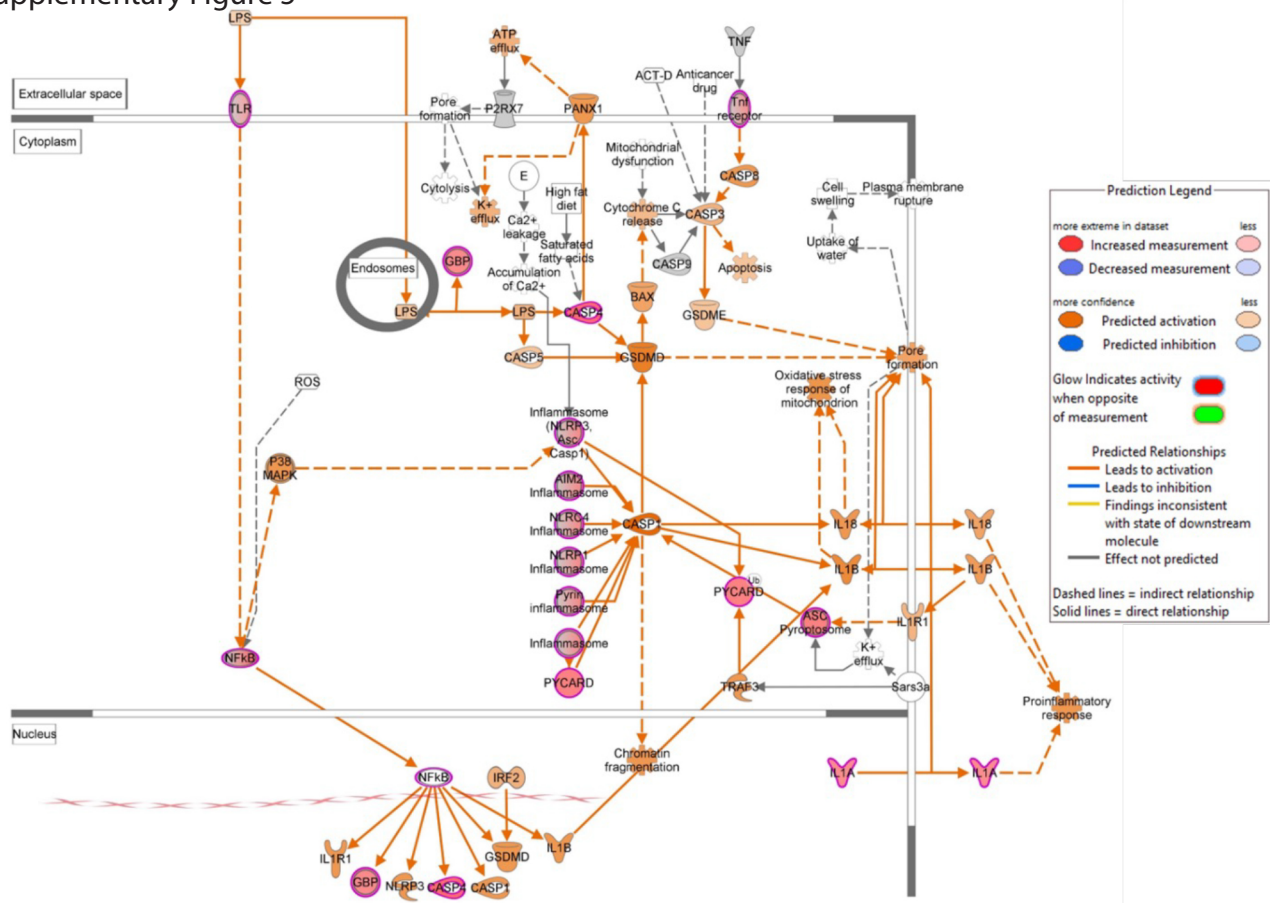

Supplement: Supplementary file 5 — Supplementary Data 5. Inflammasome‐driven transcriptional networks seen in the temporal lobe. [file BPA-36-e70086-s003.pdf]
